# Mechanisms of laccase-mediator treatments improving the enzymatic hydrolysis of pre-treated spruce

**DOI:** 10.1186/s13068-014-0177-8

**Published:** 2014-12-24

**Authors:** Ulla Moilanen, Miriam Kellock, Anikó Várnai, Martina Andberg, Liisa Viikari

**Affiliations:** Department of Food and Environmental Sciences, University of Helsinki, PO Box 27, Helsinki, 00014 Finland; VTT Technical Research Centre of Finland, PO Box 1000, Espoo, 02044 Finland; Department of Chemistry, Biotechnology and Food Science, Norwegian University of Life Sciences, PO Box 5003, Aas, N-1432 Norway

**Keywords:** Enzymatic hydrolysis, Laccase, Mediator, Lignin, Cellulose oxidation, Spruce

## Abstract

**Background:**

The recalcitrance of softwood to enzymatic hydrolysis is one of the major bottlenecks hindering its profitable use as a raw material for platform sugars. In softwood, the guaiacyl-type lignin is especially problematic, since it is known to bind hydrolytic enzymes non-specifically, rendering them inactive towards cellulose. One approach to improve hydrolysis yields is the modification of lignin and of cellulose structures by laccase-mediator treatments (LMTs).

**Results:**

LMTs were studied to improve the hydrolysis of steam pre-treated spruce (SPS). Three mediators with three distinct reaction mechanisms (ABTS, HBT, and TEMPO) and one natural mediator (AS, that is, acetosyringone) were tested. Of the studied LMTs, laccase-ABTS treatment improved the degree of hydrolysis by 54%, while acetosyringone and TEMPO increased the hydrolysis yield by 49% and 36%, respectively. On the other hand, laccase-HBT treatment improved the degree of hydrolysis only by 22%, which was in the same order of magnitude as the increase induced by laccase treatment without added mediators (19%). The improvements were due to lignin modification that led to reduced adsorption of endoglucanase Cel5A and cellobiohydrolase Cel7A on lignin. TEMPO was the only mediator that modified cellulose structure by oxidizing hydroxyls at the C6 position to carbonyls and partially further to carboxyls. Oxidation of the reducing end C1 carbonyls was also observed. In contrast to lignin modification, oxidation of cellulose impaired enzymatic hydrolysis.

**Conclusions:**

LMTs, in general, improved the enzymatic hydrolysis of SPS. The mechanism of the improvement was shown to be based on reduced adsorption of the main cellulases on SPS lignin rather than cellulose oxidation. In fact, at higher mediator concentrations the advantage of lignin modification in enzymatic saccharification was overcome by the negative effect of cellulose oxidation. For future applications, it would be beneficial to be able to understand and modify the binding properties of lignin in order to decrease unspecific enzyme binding and thus to increase the mobility, action, and recyclability of the hydrolytic enzymes.

**Electronic supplementary material:**

The online version of this article (doi:10.1186/s13068-014-0177-8) contains supplementary material, which is available to authorized users.

## Background

To meet the current targets for the production of liquid fuels based on renewable sources, lignocellulosic feedstocks will have to be utilized in increasing amounts. Lignocellulosic biomass is, however, a challenging raw material because of its recalcitrant structure. It is composed mainly of structural polysaccharides that are more difficult to degrade into fermentable sugars than storage polysaccharides such as starch. The crystalline structure of cellulose makes it highly resistant to enzymatic hydrolysis. In addition, hemicelluloses and lignin form a complex network that shields cellulose from enzymatic attack [1,2]. Lignin is especially problematic, since the most common pre-treatment methods, such as steam pre-treatment, solubilize most of the hemicelluloses but leave modified lignin behind in the insoluble matrix [3]. In addition to blocking the cellulose surface from the hydrolytic enzymes, lignin is known to bind enzymes non-specifically [4-8], rendering them inactive towards cellulose, especially at hydrolysis temperatures [9].

Softwoods are an abundant source of lignocellulosic biomass in the Northern Hemisphere, and therefore their use as feedstock for liquid fuel production has aroused interest. Softwoods are, however, difficult to degrade with hydrolytic enzymes because of the structure of lignin. Softwood lignin is largely of the guaiacyl (G) type and has been shown to inhibit the enzymatic hydrolysis of cellulose more strongly than hardwood or grass lignin [10].

One way to improve the yields of the enzymatic hydrolysis of softwood would be the use of laccase-mediator treatments (LMTs) to modify the lignin and possibly the cellulose structure. Laccases (benzenediol: oxygen oxidoreductase, EC 1.10.3.2) are multi-copper oxidases able to oxidize various phenolic compounds by one electron transfer with the concomitant reduction of oxygen to water [11,12]. Laccases can only oxidize phenols and aromatic or aliphatic amines that have lower redox potential than the laccase (<0.4-0.8 V) and are small enough to enter the active center of the enzyme [13]. With the aid of low molecular weight substrate molecules as mediators, oxidation by laccases can, however, be expanded to larger molecules unable to fit into the enzymatic pocket or even to non-phenolic compounds that are not actual substrates of laccases [14,15].

Several mechanisms for the oxidation of substrates by mediators have been proposed. ABTS (2,2,’-azino-bis(3-ethylbenzothiazoline-6-sulfonic acid)) is thought to oxidize the substrate by an electron transfer (ET) mechanism where one electron is removed from the substrate [14,16]. N-OH type mediators such as HBT (1-hydroxybenzotriazole) are likely to act through a radical hydrogen atom transfer (HAT) route, where the mediator is oxidized into a radical that can oxidize a substrate having a higher redox potential than the mediator itself. With the HAT route, a hydrogen atom is transferred from the substrate to the mediator, as opposed to the ET route where only the electron is transferred to the mediator and the H^+^ ion from the substrate is released into the medium [17,18]. Oxidation with TEMPO (2,2,6,6-tetramethylpiperidine-1-oxyl) is understood to differ from these two reactions and involve an ionic mechanism. TEMPO is a stable N-oxyl radical that is oxidized to a reactive oxoammonium ion by laccase. The oxoammonium ion is proposed to oxidize the primary hydroxyl via a base attack. The ionic oxidation mechanism is not dependent on the redox potential of the substrate [17,19-21].

Since the discovery of the enhancing effect of mediators, especially in lignin degradation, the use of LMTs has been studied for many applications in lignocellulosics, such as pulp bleaching and refining as well as other fiber modifications (reviewed by Widsten and Kandelbauer [22]). In addition, LMTs have been used in several other application areas; in organic synthesis LMTs can catalyze diverse reactions, and in waste water treatment they can detoxify or remove xenobiotic compounds, such as textile dyes and chlorophenols (reviewed in [23-25]). In recent years, LMT research has focused on finding natural mediators to replace synthetic ones [26]. Natural mediators can be either fungal phenolic metabolites or lignin-derived phenols [27,28]. The advantage of natural mediators is that they may be less toxic and that they could be produced at a lower cost than synthetic ones. In addition, some can be available in the lignocellulosic raw material [26].

**Figure 1:**
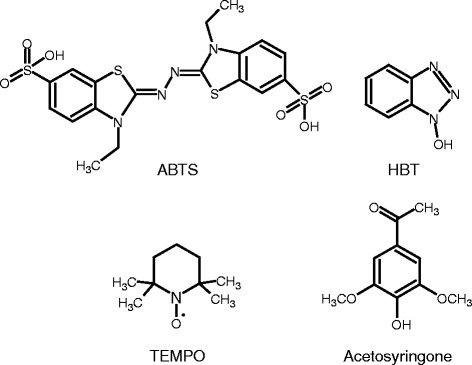
**Structures of the mediators used in the study.**

In this paper, LMTs were studied to improve the hydrolysis of pre-treated spruce. Three mediators with three distinct reaction mechanisms (ABTS, HBT, and TEMPO) and one natural mediator (AS, that is, acetosyringone) were tested. The structures of the mediators are shown in Figure 1. Laccase-mediator systems have generally been targeted to act specifically on the lignin moiety of the lignocellulosic substrates. Thus, their possible impacts on cellulose and therefore on enzymatic cellulose hydrolysis have been insufficiently studied. In this study, the effect of LMT on both cellulose and lignin fractions was investigated.

## Results and discussion

### The effect of LMTs on the enzymatic hydrolysis of steam pre-treated spruce

**Figure 2:**
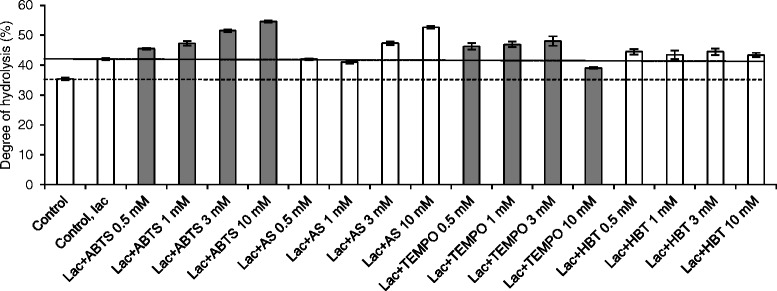
**Enzymatic hydrolysis of thermochemically pre-treated spruce after treatments with laccase and various mediators.** Error bars represent the standard errors of the means of triplicate experiments.

To improve the degree of enzymatic hydrolysis of steam pre-treated spruce (SPS), the substrate was treated with *Trametes hirsuta* laccase alone or in combination with one of the mediators ABTS, HBT, TEMPO, or AS prior to enzymatic hydrolysis. The LMTs were studied at various mediator concentrations (0.5, 1, 3, and 10 mM). Laccase treatment alone increased hydrolysis by 19% compared with the reference, which was not treated by laccase (Figure 2). This increase is in the same order of magnitude as reported in previous studies, where laccase treatment without added mediators improved the enzymatic hydrolysis of SPS by 12 to 13% [29,30].

In this study, all LMTs improved the enzymatic hydrolysis of SPS. Notably, of the tested laccase and mediator combinations, the laccase and ABTS treatment gave the most marked improvement in the degree of hydrolysis. A 54% increase in conversion was observed when laccase and 10 mM of ABTS were used. Similarly, AS was found to be an effective laccase mediator in higher doses; when loaded at 10 mM concentration, it provided an increase of 49% compared with the reference. TEMPO also improved enzymatic conversion of SPS to sugars; 3 mM TEMPO enhanced the hydrolysis by 36%. When TEMPO was used at 10 mM concentration, however, the hydrolysis yield was impaired since the laccase treatment alone led to higher yields of released sugars. Surprisingly, the use of HBT did not enhance the degree of hydrolysis further.

There are only a few studies where LMTs have been used to improve the enzymatic hydrolysis of lignocellulose-containing substrates. Palonen and Viikari [30] used *T. hirsuta* laccase with N-hydroxy-N-phenylacetamide (NHA) to treat steam pre-treated softwood prior to enzymatic hydrolysis and gained up to 21% improvement in the hydrolysis yield. The positive effect was considered to be due to the removal of lignin, but could also result from an expected modification of the surface lignin structure affecting enzyme-substrate interaction. In another study by Gutiérrez *et al.* four sequential laccase-HBT treatments followed by alkaline peroxide extraction of eucalyptus and elephant grass increased glucose yield by 61% with eucalyptus and 12% with elephant grass compared with those without enzymatic treatment [31]. The improvement of hydrolysis was attributed to a decrease of 34% and 22% in the lignin contents of eucalyptus and elephant grass, respectively. In addition, changes in the lignin structure were observed as a result of the laccase-HBT treatment. The share of G units appeared to decrease to a higher extent than that of the syringyl (S) units, leading to residual lignin consisting mostly of oxidized S units. In a further study by the same authors, similar improvements on the enzymatic hydrolysis of eucalyptus was gained when the *Trametes villosa* laccase was replaced with a recombinant *Myceliophthora thermophila* laccase and the synthetic HBT mediator was changed to a natural mediator; methyl syringate [32]. Heap *et al.*, on the other hand, used laccase-HBT treatment in combination with alkaline peroxide extraction to improve (by 35%) the saccharification yield of acid pre-treated wheat straw. It was observed that the LMT impaired the hydrolysis yield when not combined with the alkaline extraction step. The authors concluded that lignin extraction was enhanced by the LMT-induced formation of Cα oxidized groups in lignin [33].

### Cellulose oxidation with LMTs

**Figure 3:**
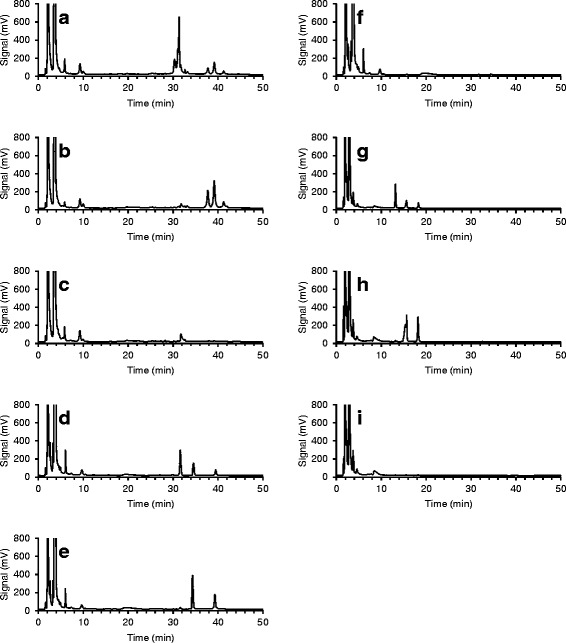
**Analysis of the oxidation products of phosphoric acid swollen cellulose by HPAEC-PAD after laccase-TEMPO treatment. (a)** Laccase-TEMPO treatment (LTT) and enzymatic hydrolysis (EH); **(b)** LTT, NaClO_2_ oxidation, and EH; **(c)** TEMPO treatment, NaClO_2_ oxidation, and EH; **(d)** LTT, EH, and acid hydrolysis (AH); **(e)** LTT, NaClO_2_ oxidation, EH, and AH; **(f)** TEMPO treatment, NaClO_2_ oxidation, EH, and AH; **(g)** LTT, EH, and AH; **(h)** LTT, NaClO_2_ oxidation, EH, and AH; **(i)** TEMPO treatment, NaClO_2_ oxidation, EH, and AH. **(a-c)** Diluted 1:5; **(d-f)** diluted 1:2; **(a-f)** eluted with gradient 1; **(g-i)** eluted with gradient 2.

To study the possible modification of cellulose structure by LMTs, phosphoric acid swollen cellulose (PASC) was treated with laccase and mediators (ABTS, HBT, TEMPO, or AS at 10 mM concentration). Amorphous PASC was used as a model substrate because of its higher surface area and accessibility to oxidative reactions compared with the highly crystalline Avicel. To decrease the degree of polymerization and to solubilize the products, the treated PASC samples were enzymatically hydrolyzed. The hydrolyzed oxidation products were then analyzed with high-performance anion exchange chromatography with pulsed amperometric detection (HPAEC-PAD). Of the mediators examined, only TEMPO applied together with laccase produced peaks not found in the control samples. Thus, laccase-TEMPO treatment was the only treatment that oxidized PASC, suggesting that of the three possible mediated oxidation mechanisms, only the ionic oxidation mechanism was able to oxidize cellulose. After the laccase-TEMPO treatment followed by enzymatic hydrolysis, several unidentified elution peaks were observed in the chromatogram at 30 to 33 min and at 37 to 42 min (Figure 3a) when eluted with gradient 1 (Table 1). In an attempt to identify these oxidation products, the expected carbonyl (aldehyde) groups formed during laccase-TEMPO treatment were further oxidized to carboxyl groups by NaClO_2_ oxidation. Laccase-TEMPO treatment is known to oxidize the primary hydroxyl groups of cellulose to carbonyl and partially further to carboxyl groups at the C6 position, yielding 6-aldehydo-D-glucose and D-glucuronic acid units [34]. These compounds are further oxidized by NaClO_2_; the available free carbonyl groups, that is, the carbonyl group at the C6 position of the 6-aldehydo-D-glucose and the carbonyl group of the anomeric carbon (C1) of the D-glucose unit at the reducing end, are converted to carboxyl groups, yielding D-glucuronic acid and D-gluconic acid, respectively (Additional file 1: Figure S1). NaClO_2_ oxidation is known to oxidize carbonyl groups to carboxyl groups selectively, without oxidizing primary hydroxyls (at the C6 carbon) to carbonyls [35].

**Table 1:** **The gradients used in the HPAEC-PAD analysis for oligosaccharides**

**Gradient 1**			**Gradient 2**		
**Time (min)**	**A (%)**	**B (%)**	**Time (min)**	**A (%)**	**B (%)**
0	0	100	0	2	98
15	0	100	30	30	70
35	12	88	35	30	70
40	12	88	40	2	98
45	0	100	50	2	98
50	0	100			

A: 1 M NaAc in 100 mM NaOH; B: 100 mM NaOH.

After NaClO_2_ oxidation, the peaks in the enzymatically hydrolyzed samples exhibited a clear shift from 30 to 33 min to 37 to 42 min, indicating that peaks eluting at 30 to 33 min represented compounds with carbonyl groups and peaks eluting at 37 to 42 min represented compounds with the corresponding carboxyl groups (Figure 3b). No such peaks were found in the laccase-free control (Figure 3c), which confirms that they were not produced by TEMPO alone, by NaClO_2_ oxidation of glucose units, or by enzymatic hydrolysis. Recently, Patel *et al.* [36] also studied the oxidation of cotton linters pulp with various LMTs testing ABTS, HBT, TEMPO, violuric acid, and promazine as mediators for laccase. In agreement with the present study, it was found that only laccase-TEMPO treatment caused oxidative modification of cellulose. Selective labeling in combination with gel permeation chromatography was used to identify the oxidation products. It was concluded that the oxidized groups in the pulp were mostly carbonyl groups but carboxyl groups were also found. The results of the present study on the HPAEC-PAD analysis of the oxidized products of PASC by laccase-TEMPO treatment support these findings.

Oxidized groups in cellulose can prevent cellulases, especially cellobiohydrolases and β-glucosidases, from completely monomerizing cellulose. Therefore in the present study, the enzymatic hydrolysates were further hydrolyzed with mild acid to break down any possible oligomeric compounds (containing carbonyl or carboxyl groups) into monomeric units. Analysis of the oxidation products (after the enzymatic and mild acid hydrolysis of laccase-TEMPO and NaClO_2_-treated PASC samples) confirmed the formation of D-glucuronic acid eluting at 34 min when using HPAEC-PAD with gradient 1 (Figure 3d and e), as confirmed by standards (Additional file 2: Figure S2). The concentration of D-glucuronic acid in the sample treated by laccase-TEMPO was 1.16 μmol ml^−1^, and after further oxidation with NaClO_2_ the concentration increased to 3.16 μmol ml^−1^, corresponding to 2.6% of the total amount of glucose units, or on average every 40th glucose unit in cellulose being oxidized. As the degree of polymerization of Avicel (and thus of PASC) is in the range of 100 to 300 units, cellulose chains contained more than one oxidation site per polymer/cellulose chain. Furthermore, it can be anticipated that the oxidation of the primary hydroxyls happened at the most accessible areas of cellulose microfibrils, namely at the non-reducing ends of the cellulose chains and on cellulose chains located at the surface of cellulose microfibrils, where the oxoammonium ion had easy access.

After the mild acid hydrolysis of PASC treated with laccase-TEMPO and cellulases (but not with NaClO_2_), three significant peaks were detected (Figure 3d). One eluted at 34 min, identified as D-glucuronic acid; for the identification of the two other peaks, eluting at 31 min and 39 min, standards were lacking. After the NaClO_2_ oxidation, the height (and area) of the peaks of D-glucuronic acid (at 34 min) and of the one appearing at 39 min increased considerably, and the peak at 31 min disappeared (Figure 3e). This indicates that the 31-min peak was 6-aldehydo-D-glucose being oxidized to D-glucuronic acid (34 min) with NaClO_2_. Furthermore, the increase in the size of the third peak (at 39 min) indicates that it was a compound with a higher degree of oxidation. None of these oxidation products was present in the laccase-free control (Figure 3f).

To identify the unknown peak eluting at 39 min, the NaClO_2_-oxidized samples were analyzed again with the HPAEC-PAD system with gradient 2 (Table 1 and Figure 3 g-i). This time a new peak appeared at around 8 min and was identified by a standard as D-gluconic acid (Additional file 2: Figure S2). With this type of analysis, the D-gluconic acid peak was flat and wide and therefore difficult to detect. By altering the gradient, the D-gluconic acid peak could be detected more accurately in samples subjected to enzymatic and mild acid hydrolysis after laccase-TEMPO treatment (Figure 3g, around 20 min in Figure 3d). However, the signal-to-noise ratio was too low to confirm unambiguously the formation of D-gluconic acid by laccase-TEMPO treatment. On the other hand, D-gluconic acid was clearly formed by chemical oxidation (Figure 3h and i). NaClO_2_ oxidized not only the 6-aldehydo-D-glucose to D-glucuronic acid but also the unprotected C1 carbonyl at the reducing end of the cellulose chain to D-gluconic acid. Notably, when the samples were eluted with gradient 2, the peak assigned to D-glucuronic acid (15 min, Figure 3h) split into two overlapping peaks, indicating that another compound co-eluted. L-guluronic acid is expected to elute very closely to D-glucuronic acid on the HPAEC-PAD column due to their similar structures (Additional file 1: Figure S1). If the laccase-TEMPO treatment oxidized glucose units located at the reducing end of cellulose to 6-aldehydo-D-glucose, then two products could be formed upon further oxidization: D-glucuronic acid (6-aldehydo-D-glucose oxidized at the C6 position) and L-guluronic acid (6-aldehydo-D-glucose oxidized at the C1 position). In fact, oxidation at the reducing end could also explain the third unassigned peak (39 min in Figure 3d and e or 18 min in Figure 3g and h), which would then be dicarboxylic acid, that is, D-glucaric acid, being formed at the reducing end by further oxidation of the carbonyl group of either D-glucuronic acid or L-guluronic acid to a carboxylic group.

In conclusion, of the mediators studied, only TEMPO was able to oxidize PASC when combined with laccase. The possible oxidation products of D-glucose units by laccase-TEMPO treatment are shown in Additional file 1: Figure S1. Laccase-TEMPO treatment of PASC oxidized D-glucose units primarily at the C6-position, mostly at the non-reducing ends of the cellulose chain and on the surface of cellulose microfibrils, forming 6-aldehydo-D-glucose. In addition, some of these aldehydes were further oxidized to D-glucuronic acid. Furthermore, the results indicate that laccase-TEMPO treatment can lead to the oxidation of reducing end D-glucose units at the C6 position, allowing NaClO_2_ to oxidize the 6-aldehydo-D-glucose unit further to D-glucuronic, L-guluronic, and D-glucaric acids. Chromatographic data suggests the formation of D-gluconic acid and D-glucaric acid (Figure 3d and g) by oxidation solely with laccase-TEMPO treatment. Accordingly, the oxidation of free carbonyl groups of the anomeric carbon at the reducing end of cellulose to carboxyl groups by laccase-TEMPO treatment is also likely and cannot be excluded, as the commercial cellulase preparation used (Celluclast 1.5L) lacks oxidative cellulose-degrading enzymes.

**Figure 4:**
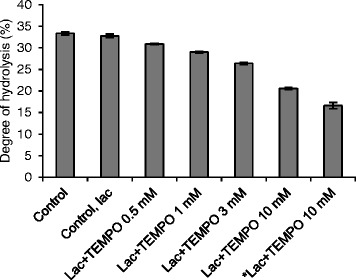
**Enzymatic hydrolysis of microcrystalline cellulose (Avicel) after treatment with laccase and TEMPO.** * = Samples washed three times with 5 ml of ultrapure water prior to enzymatic hydrolysis. Error bars represent the standard errors of the means of triplicate experiments.

To study the impact of the cellulose oxidation on the enzymatic hydrolysis of cellulose, Avicel was treated with laccase and TEMPO and further hydrolyzed with the commercial cellulase preparation (Figure 4). Notably, on the pure cellulose substrate Avicel, a low mediator concentration already had an adverse effect on the degree of hydrolysis. Increasing the mediator concentration impaired the degree of hydrolysis, obviously due to a growing number of oxidation sites. When the concentration of TEMPO was increased to 10 mM the degree of hydrolysis declined from 33 to 21%. The formation of carbonyl and carboxyl groups on Avicel can be expected to especially inhibit the action of cellobiohydrolases and β-glucosidases, as they act on chain ends and cellooligomers, respectively. In addition, inter-fiber covalent bonds through hemiacetal linkages between hydroxyl groups and carbonyl groups may have been formed after the laccase-TEMPO treatment, increasing the strength of the cellulose [35]. To confirm that the inhibition of the hydrolysis was caused by cellulose oxidation and not by the interaction of oxidized TEMPO with cellulases, an additional experiment was performed: Avicel, oxidized by laccase and (10 mM) TEMPO, was washed three times with 5 ml of ultrapure water prior to the enzymatic hydrolysis step to remove residual laccases and oxidized TEMPO that might affect the performance of the hydrolytic enzymes. Again, the hydrolysis yield was reduced from 33 to 17%, verifying that cellulose oxidation was the cause of the hydrolysis impairment (Figure 4).

Thus, it is significant that even though the treatment of Avicel by 3 mM laccase-TEMPO clearly inhibited hydrolysis, the degree of hydrolysis was improved when SPS was used as a substrate. These results indicate that the positive effects on lignin caused by the treatment outweighed the negative effects on cellulose or that the oxidative systems preferably attacked lignin. When the TEMPO concentration was increased to 10 mM on SPS, the oxidation of cellulose was the determining factor in reducing the degree of hydrolysis. Previously, the oxidation of cellulose by lytic polysaccharide monooxygenases has been observed to improve the hydrolysis of cellulose. It has been concluded that the positive effect is due to the oxidation of the C1 or C4 position in the D-glucose units, causing the cleavage of the β-1,4 linkage in the cellulose chain [37]. Notably, as opposed to the laccase-TEMPO treatment, the action of lytic polysaccharide monooxygenases leads to the formation of two new cellulose chain ends, one oxidized and one non-oxidized, increasing the number of sites available for the action of cellobiohydrolases.

### Effect of LMTs on SPS lignin

As the oxidation of cellulose hinders the hydrolytic action of cellulases (Figure 4), the positive effects of oxidative treatments on SPS hydrolysis (Figure 2) can be expected to have been caused by the modification of lignin. Cellulases are known to adsorb non-specifically to lignin [4], and thus oxidative modification may affect the binding properties of lignin. The adsorption properties of a mixture of purified enzymes (70% *Trichoderma reesei* Cel7A, 25% *T. reesei* Cel5A, and 5% *Aspergillus niger* Cel3A) to isolated SPS lignin were assessed using the Langmuir isotherm (Eq. 1). Cel7A and Cel5A are among the main components in Celluclast 1.5L, whereas Cel3A is the β-glucosidase present in Novozym 188. The maximum adsorption capacity of SPS lignin was 55 mg protein g^−1^ lignin, the affinity constant was 4.0 ml mg^−1^, and the binding strength was 220 ml g^−1^ lignin. These values are somewhat higher than previously reported for spruce lignin. For example, Rahikainen *et al.* [38] determined the Langmuir isotherms for similarly treated and isolated spruce lignin using *Melanocarpus albomyces* Cel45A endoglucanase fused with a linker and a carbohydrate binding module from *T. reesei* Cel7A. In that study, the maximum adsorption capacity was 42 mg protein g^−1^ lignin, the affinity constant 1.5 ml mg^−1^, and the binding strength 64 ml g^−1^ lignin. Previously, adsorption experiments with Cel45A were performed at 4°C, whereas the enzyme mixture used in this study was adsorbed at 45°C. Adsorption of Celluclast 1.5L on isolated spruce lignin has been reported to increase when the temperature is raised from 4°C to 45°C. Furthermore, the enzymes have been observed to have stronger interaction with SPS lignin at elevated temperatures [9], which would explain the differences observed in the Langmuir isotherms.

**Figure 5:**
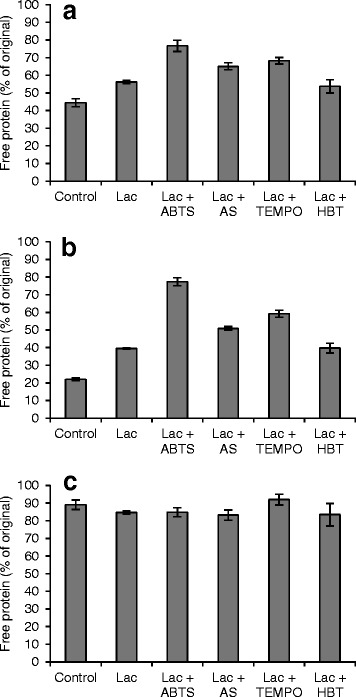
**Adsorption of purified cellulases on the isolated laccase- and mediator-treated SPS lignins. (a)** Cellobiohydrolase Cel7A, **(b)** endoglucanase Cel5A, and **(c)** β-glucosidase Cel3A. Error bars represent the standard errors of the means of triplicate experiments.

To study the effects of various oxidative treatments by laccase and mediators on the non-specific binding of the enzymes on lignin, isolated SPS lignin was treated with laccase and 10 mM mediators. To observe clearly the differences in adsorption caused by the treatments, the concentration of the cellulase mixture used was 50 mg g^−1^, which is lower than the maximum adsorption capacity of untreated SPS lignin. Under these conditions, the untreated SPS lignin bound more than half of the cellobiohydrolase Cel7A, leaving 44% of the proteins free in the solution (Figure 5a). Treating SPS lignin with laccase alone decreased the binding of Cel7A by 27%. The adsorption of Cel7A was further decreased by supplementing a mediator. Of the mediators used, ABTS changed the binding properties of the SPS lignin most considerably, increasing the share of free Cel7A to 77%. AS and TEMPO also decreased the non-specific binding of Cel7A on lignin: after the treatments, 65% and 68% of the protein remained unbound, respectively. Notably, the impact caused by the oxidative treatments was even more substantial on the endoglucanase Cel5A (Figure 5b). The Cel5A was bound to the untreated lignin to a higher degree than Cel7A. The same phenomenon has also been observed previously with *T. reesei* cellulases [29]. The laccase treatment changed the binding properties of the lignin by increasing the percentage of free Cel5A from 22 to 40%. As with Cel7A, the laccase-ABTS treatment had the most notable effect on the binding of Cel5A by increasing the share of free enzyme to 77%. Again, the laccase-TEMPO and laccase-AS treatments also decreased the adsorption of Cel5A compared with the control. On the other hand, laccase-HBT treatment of lignin did not affect the binding of any of the enzymes in the mixture compared with the mediator-free laccase control. As anticipated, the adsorption of β-glucosidase did not change after the treatments (Figure 5c), since most of the enzymes remained free even after incubation with untreated SPS lignin. Observations on the binding of cellulases on lignin after LMT have not been previously described.

The inability of the laccase-HBT treatment to improve the hydrolysis of SPS (Figure 2) was explained by the unchanged binding properties of laccase-HBT treated lignin (Figure 5), and raised the question of whether HBT is a suitable substrate for the *T. hirsuta* laccase. To confirm that *T. hirsuta* laccase was able to oxidize HBT, the oxygen consumption of laccase and HBT was measured. It was observed that oxygen was consumed 40 times more slowly with HBT than with the other mediators (Additional file 3: Figure S3). In other words, HBT was not an optimal substrate for *T. hirsuta* laccase. Bourbonnais *et al.* [39] noticed the same phenomenon when the oxygen consumption of *Trametes versicolor* laccase was measured with ABTS or HBT. It was reported that the oxidation of HBT by laccase was more than 85 times slower than the oxidation of ABTS. In addition, it was observed that during pulp delignification, HBT inactivated the *T. veriscolor* laccase almost completely, whereas when using ABTS, 32% of the initial laccase activity was recovered.

**Figure 6:**
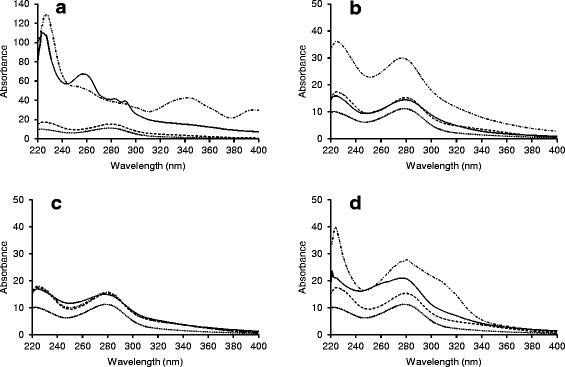
**The modification of soluble aromatic compounds of SPS caused by laccase-mediator treatment.** UV absorbance spectrum (220 to 400 nm) was measured from the liquid fractions of enzymatically hydrolyzed SPS samples treated with laccase and 3 mM mediators. Mediators used were **(a)** ABTS, **(b)** AS, **(c)** TEMPO, and **(d)** HBT. Dashed line = reference enzymatic hydrolysis (lacking laccase-mediator treatment), dotted line = reference laccase treatment followed by enzymatic hydrolysis (lacking mediator), dash-dotted line = combined curve of reference enzymatic hydrolysis (lacking laccase-mediator treatment) and reference laccase and mediator (lacking SPS), and solid line = laccase-mediator treatment followed by enzymatic hydrolysis.

In addition to the ability of LMTs to modify the cellulase binding properties of lignin, the treatments may also have changed the lignin contents of the treated samples. To study these changes, both soluble and insoluble lignins were analyzed. The modifications of soluble aromatic compounds were detected by measuring the UV absorbance spectrum (220 to 400 nm) from the liquid fractions of enzymatically hydrolyzed SPS samples treated first with laccase and 3 mM mediators (Figure 6). Enzymatic hydrolysates of SPS without LMT or laccase-treated samples (lacking mediators) were used as reference. In addition, a combined reference curve was calculated from the reference samples of enzymatic hydrolysates of untreated SPS and samples incubated with laccase and mediator in the absence of SPS. The solid lignin content, on the other hand, was determined by the Klason lignin method from the SPS samples after the LMTs (Table 2).

**Table 2:** **Lignin content in SPS after laccase and 10 mM mediator treatments**

	**Acid insoluble lignin (%)**	**Acid soluble lignin (%)**
Control	44.4 ± 0.7	1.0 ± 0.0
Laccase	45.9 ± 0.2	0.9 ± 0.1
Laccase + ABTS	42.5 ± 0.5	1.6 ± 0.0
Laccase + AS	47.7 ± 0.6	1.5 ± 0.1
Laccase + TEMPO	43.9 ± 0.4	1.0 ± 0.1
Laccase + HBT	44.2 ± 0.2	1.3 ± 0.0

Errors calculated as the standard errors of the means of triplicate experiments.

Laccase treatment alone reduced the total amount of soluble aromatic compounds in the liquid fraction (Figure 6) and increased the lignin content of the solid fraction (Table 2), which indicates that laccase polymerized solubilized aromatic compounds on lignin. The same has been previously observed when SPS was treated with *Cerrena unicolor* laccase [29]. Of the studied mediators, ABTS, when applied together with laccase, was the only mediator able to solubilize some of the SPS lignin. According to the Klason lignin determination (Table 2), 4% of the acid insoluble lignin was solubilized, which was also visible in the UV spectra of the liquid fractions as an increase of the absorbance at 245 to 295 nm compared with the control (Figure 6a). The inability of the LMTs to solubilize significant amounts of SPS lignin can be caused by the insolubility of lignin in aqueous solutions [40]. In other words, the LMTs could potentially degrade lignin, but the fragments would not be soluble in a pH 5 buffer. Thus, LMTs are more likely to cause modifications of the surface lignin, observed as changes in the adsorptions of cellulases (Figure 5), rather than to cause lignin dissolution. It is also possible that aromatic fragments solubilized from lignin by laccase and ABTS may have acted as mediator(s) for further lignin modification employing the HAT mechanism typical for lignin-derived mediators [26], which would explain the remarkable improvements in the enzymatic hydrolysis yield (Figure 2) and the decreased enzyme adsorption (Figure 5).

Notably, laccase appeared to polymerize AS on the lignin, which was observed as an increase in the lignin content (Table 2) and as a decrease of aromatic compounds in the UV absorbance spectra (Figure 6b). In previous delignification studies using LMTs, it has been observed that natural phenolic mediators have a tendency to bind to lignin rather than to dissolve it [26]. It might be that the improvement of the enzymatic hydrolysis (Figure 2) and the change in the enzyme adsorption (Figure 5) was a result of the S-type AS covering the G-type spruce lignin surface, which thus contained a higher portion of S-type moieties after the treatment. Studer *et al.* [41] studied the enzymatic hydrolysis of 47 *Populus trichocarpa* tree samples. The trees were selected out of 1,100 individuals on the basis of the content and ratio of S and G units in lignin. They observed that the sugar yields increased with increasing S/G ratios. Furthermore, Nakagame *et al.* [10] showed that GS-type lignin isolated from poplar adsorbed fewer cellulases than G-type lignin isolated from lodgepole pine. Thus, the two types of lignins appear to have different cellulase binding properties, which was also apparent in the present study.

Laccase-HBT and laccase-TEMPO treatments did not change the amount of lignin in the solid fractions significantly (Table 2). Obviously, however, the laccase-TEMPO treatment modified the surface properties of the lignin in SPS, leading to reduced binding of cellulases (Figure 5) and, despite the adverse effect of laccase-TEMPO treatment on the enzymatic hydrolysis of cellulose (Figure 4), improved the enzymatic hydrolysis of SPS (Figure 2).

## Conclusions

The improving mechanism of LMTs on the enzymatic hydrolysis of SPS was based on the modification of the SPS lignin, resulting in decreased adsorption of cellulases on lignin and increased hydrolysis yields. On the other hand, cellulose oxidation by laccase-TEMPO treatment was observed to impair the enzymatic hydrolysis of cellulose. For future applications, it would be beneficial to be able to understand and modify the binding properties of lignin in order to decrease unspecific enzyme binding and thus to increase the mobility, action, and recyclability of the hydrolytic enzymes.

## Methods

### Raw materials

Spruce chips were impregnated with SO_2_ gas (residence time 13 min) and steam pre-treated at 212°C for 4 to 5 min. The SPS provided by Sekab E-Technology (Sweden) was washed three times with 80°C water before use. The SPS lignin was isolated as described in Moilanen *et al.* [29] by an extensive enzymatic hydrolysis [8], and the bound hydrolytic enzymes were removed with a protease treatment [42]. Microcrystalline cellulose Avicel (Fluka, Ireland) and PASC were used as cellulose model compounds. PASC was prepared from Avicel by modifying Wood’s method [43]. Avicel (25 g) was slowly added to 400 ml of 85% (V V^−1^) phosphoric acid at 4°C, as the mixture was blended in a kitchen homogenizer. The solution was incubated at 4°C overnight. PASC was extensively washed with ultrapure water until the pH of the supernatant was 5. The last wash was performed with 100 mM sodium acetate buffer, pH 5, and the PASC was stored at 4°C.

### Enzymes and mediators

Laccase from *T. hirsuta* was produced and purified as described in Rittstieg *et al.* [44]. The hydrolytic enzymes used were cellulases from *T. reesei* (Celluclast 1.5L, Novozymes, Denmark) and β-glucosidase from *A. niger* (Novozym 188, Novozymes, Denmark). The monocomponent cellulases cellobiohydrolase Cel7A (EC 3.2.1.91) and endoglucanase Cel5A (EC 3.2.1.4) from *T. reesei* were purified according to Suurnäkki *et al.* [45] and the β-glucosidase Cel3A (EC 3.2.1.21) from *A. niger* by the method described in Sipos *et al.* [46]. Four commercial compounds - TEMPO (Aldrich, Poland), HBT (Sigma, Japan), ABTS (Sigma, Canada), and AS (Aldrich, India) - were used as mediators. The mediators were dissolved in 100 mM sodium acetate buffer-ethanol solution (1:1 V V^−1^). A fresh batch of the mediator solutions was prepared for each experiment.

### Laccase activity assay

Laccase activity was determined using ABTS as substrate, according to Niku-Paavola *et al.* [47].

### Determination of protein concentration

Protein concentration was determined by the Lowry method [48] (absolute protein concentrations) or with gel electrophoresis (relative protein concentrations) using the Criterion Stain Free Imager (Bio-Rad, USA) system described in Várnai *et al.* [49]. With the Lowry method, interfering substances were eliminated by precipitating the proteins with acetone (1:4 ratio of protein solution to acetone). The precipitate was dissolved in a solution containing Na_2_CO_3_ (2%) and NaOH (0.4%) before measurement. Bovine serum albumin (Sigma, USA) was used as the standard in the Lowry method, while the mixture of pure enzymes was used as the standard in the quantification with gel electrophoresis.

### Carbohydrate analysis

Monosaccharides were determined with the HPAEC-PAD system as described by Moilanen *et al.* [29]. The cellulose oxidation products were also analyzed with an HPAEC-PAD system according to the method described by Rantanen *et al.* [50] for analysis of oligosaccharides. The eluents for gradient analysis of the oxidation products were A: 1 M NaAc in 100 mM NaOH and B: 100 mM NaOH. The samples were analyzed with two different gradients named gradient 1 and gradient 2 (Table 1). D-Gluconic acid sodium salt (Sigma, France), D-glucuronic acid (Sigma, Switzerland), and a cellooligosaccharide standard containing cellobiose, cellotriose, and cellotetraose (Merck, Germany) were used as standards.

### Determination of lignin content

The dissolved SPS lignin was determined by measuring the UV absorption spectrum (220 to 400 nm) spectrophotometrically from liquid samples, whereas the solid SPS lignin was determined by the Klason lignin method according to Sluiter *et al.* [51]. In this method, the samples were hydrolyzed with sulfuric acid and the acid insoluble lignin was determined from the solid residue, while the acid soluble lignin was measured from the hydrolysate spectrophotometrically at 240 nm using an absorptivity of 30 l (g cm)^−1^ [52,53].

### LMTs and enzymatic hydrolysis

LMTs were performed on SPS and Avicel at a substrate consistency of 2% (w V^−1^) dry matter (DM), in 100 mM sodium acetate buffer, pH 5, in 2 ml reaction volume, at 45°C, and 250 rpm shaking for 24 h. Laccase was added to a dosage of 1,000 nkat g^−1^ DM and the mediator concentrations were 0.5, 1, 3, and 10 mM. Untreated, laccase-treated, and mediator-treated samples were used as controls. After the treatments, laccase activity was terminated by boiling (10 min), and the hydrolytic enzymes Celluclast 1.5L (10 mg g^−1^ DM) and Novozym 188 (500 nkat g^−1^ DM) were added, together with NaN_3_ (0.02% (w V^−1^) final concentration) to avoid microbial contamination. The hydrolysis was continued for 24 h. Liquid fractions containing the released sugars were separated from solid residues by centrifugation. The released sugars were analyzed with HPAEC-PAD and the results were calculated as the degree of hydrolysis (%) of the theoretical carbohydrate yield. All the hydrolysis experiments were run in triplicate. The values reported are the means of the triplicate experiments, and the errors were calculated as the standard errors of the means.

To detect the dissolved SPS lignin, the UV absorption spectrum (220 to 400 nm) was measured spectrophotometrically from the liquid fractions of enzymatically hydrolyzed samples treated first with laccase and 3 mM mediators. Mediators oxidized by laccase without substrate were used as controls. In addition, the solid lignin content was determined from SPS samples treated with laccase and 10 mM mediators (but not with hydrolytic enzymes). Untreated and laccase-treated samples were used as controls. The values reported are the means of triplicate experiments, and the errors were calculated as the standard errors of the means.

### Cellulose oxidation

Cellulose oxidation was studied using PASC as the substrate. LMTs were carried out as described in the previous section. The laccase dosage used was 5,000 nkat g^−1^ DM, and the mediator concentration was 10 mM. To identify the carbonyl groups formed in LMT, some of the LMT samples were further oxidized chemically to the corresponding carboxyls using a method modified from Saito and Isogai [35] by adding 0.2 ml of 2 M NaClO_2_, 0.4 ml of 5 M acetic acid, and 0.4 ml of ultrapure water to washed LMT PASC. The oxidation reaction was carried out for 48 h at room temperature (23°C) in tubes with magnetic stirring. After incubation, the modified cellulose was washed three times with 5 ml ultrapure water and separated from the liquid fraction by centrifugation.

For analysis of the oxidized products, samples treated by laccase and mediators and by laccase and mediators and NaClO_2_ oxidation were enzymatically hydrolyzed into soluble sugars with Celluclast 1.5L (40 mg g^−1^ DM of substrate) and Novozym 188 (1,000 nkat g^−1^ DM). The hydrolysis was carried out as described in the previous section. Some of the samples were further hydrolyzed by dilute acid hydrolysis modified from Sluiter *et al.* [51] by adding 2 ml of sample and 100 μl of 70% H_2_SO_4_ to a 5-ml volumetric flask. The samples were autoclaved for 1 h at 120°C, and after cooling the pH was adjusted to 7 and the volume to 5 ml. Untreated, mediator-treated, laccase-treated, and NaClO_2_-oxidized PASC were used as controls.

### Adsorption experiments

Protein adsorption experiments were performed on the isolated SPS lignin treated with laccase and mediators. The treatment was carried out at a lignin concentration of 1% (w V^−1^) DM (corresponding to 2% DM of SPS), in 100 mM sodium acetate buffer, pH 5, at 45°C, and 250 rpm shaking for 24 h. Laccase was added to a dosage of 2,000 nkat g^−1^ DM, and the mediator concentration was 10 mM. After the treatments, laccase activity was terminated by boiling (10 min), and solids were separated by centrifugation and washed three times with pH-adjusted ultrapure water (pH adjusted to 2.5 with HCl). Untreated and laccase-treated lignins were used as controls and were subjected to the same treatment conditions as the LMT lignins. All lignins were lyophilized and stored at room temperature.

The adsorption was carried out at a lignin concentration of 1% (w V^−1^) DM, in 100 mM sodium acetate buffer, in a reaction volume of 1.5 ml, pH 5, at 45°C, and 250 rpm shaking for 90 min. The lignins were incubated with a mixture of purified enzymes, which contained (on a weight basis) 70% *T. reesei* Cel7A, 25% *T. reesei* Cel5A, and 5% *A. niger* Cel3A. Controls lacking lignins or enzymes were used. The free proteins in the samples were quantified with gel electrophoresis using Bio-Rad’s Criterion Stain Free Imager system.

To determine the adsorption parameters, the untreated SPS lignin was incubated with 10–200 mg g^−1^ DM of the enzyme mixture. The adsorption parameters were determined by the non-linear regression of the adsorption data using the Langmuir isotherm:

1 $$ {P}_a={P}_{a, max}\frac{K_P{P}_f}{1+{K}_P{P}_f} $$

where *P*_*a*_ is the amount of adsorbed protein (mg protein g^−1^ substrate), *P*_*a,max*_ is the maximum adsorption capacity (mg protein g^−1^ substrate), *K*_*p*_ is the affinity constant (ml mg^−1^ protein), and *P*_*f*_ is the concentration of free protein (mg protein ml^−1^). The binding strength (ml g^−1^ substrate) is defined as:

2 $$ S={P}_{a, max}{K}_P $$

For the laccase-treated and LMT lignins the enzyme mixture concentration used was 50 mg g^−1^ DM. The values reported are the means of triplicate samples, and the errors were calculated as the standard errors of the means.

## Additional files

Additional file 1: Figure S1. The oxidation of D-glucose units of cellulose by laccase-TEMPO treatment. Additional file 2: Figure S2. Standards for HPAEC-PAD analysis. (a) Glucuronic acid eluted with gradient 1, (b) gluconic acid eluted with gradient 1, (c) glucuronic acid eluted with gradient 2, and (d) gluconic acid eluted with gradient 2. Additional file 3: Figure S3. Oxygen consumption of *Trametes hirsuta* laccase (10 nkat ml^−1^) and 0.5 mM mediator: HBT (blue line), ABTS (red line), TEMPO (green line), and AS (purple line). Measured for (a) 20 min and (b) 12 h.

## Abbreviations

ABTS: 2,2,’-azino-bis(3-ethylbenzothiazoline-6-sulfonic acid)
AS: Acetosyringone
DM: Dry matter
ET: Electron transfer
G: Guaiacyl
HAT: Hydrogen atom transfer
HBT: 1-hydroxybenzotriazole
HPAEC-PAD: High-performance anion exchange chromatography with pulsed amperometric detection
LMT: Laccase-mediator treatment
NHA: N-hydroxy-N-phenylacetamide
PASC: Phosphoric acid swollen cellulose
S: Syringyl
SPS: Steam pre-treated spruce
TEMPO: 2,2,6,6-tetramethylpiperidine-1-oxyl
