# Supplementary material for: Mechanisms of laccase-mediator treatments improving the enzymatic hydrolysis of pre-treated spruce
Source: Biotechnol Biofuels. 2014 Dec 24;7:177. doi: 10.1186/s13068-014-0177-8 (PMC4297466; doi:10.1186/s13068-014-0177-8)
Supplement: Additional file 1: Figure S1. — The oxidation of D-glucose units of cellulose by laccase-TEMPO treatment. [file 13068_2014_177_MOESM1_ESM.pdf]

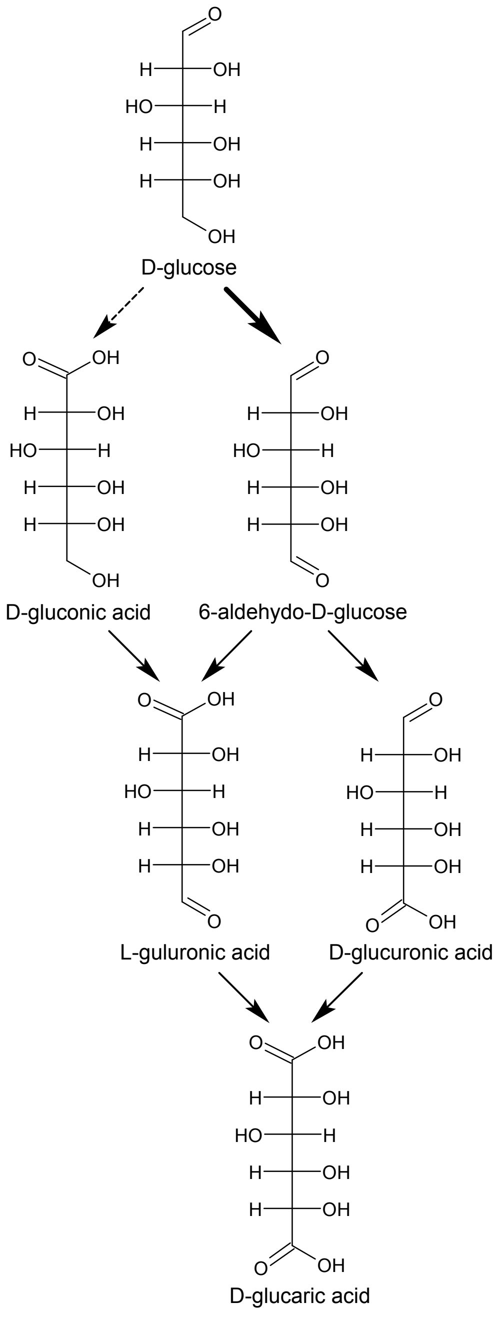

Additional file 1: Figure S1. The oxidation of D-glucose units of cellulose by laccase-TEMPO treatment.
