# Supplementary material for: Mechanisms of laccase-mediator treatments improving the enzymatic hydrolysis of pre-treated spruce
Source: Biotechnol Biofuels. 2014 Dec 24;7:177. doi: 10.1186/s13068-014-0177-8 (PMC4297466; doi:10.1186/s13068-014-0177-8)
Supplement: Additional file 2: Figure S2. — Standards for HPAEC-PAD analysis. (a) Glucuronic acid eluted with gradient 1, (b) gluconic acid eluted with gradient 1, (c) glucuronic acid eluted with gradient 2, and (d) gluconic acid eluted with gradient 2. [file 13068_2014_177_MOESM2_ESM.pdf]

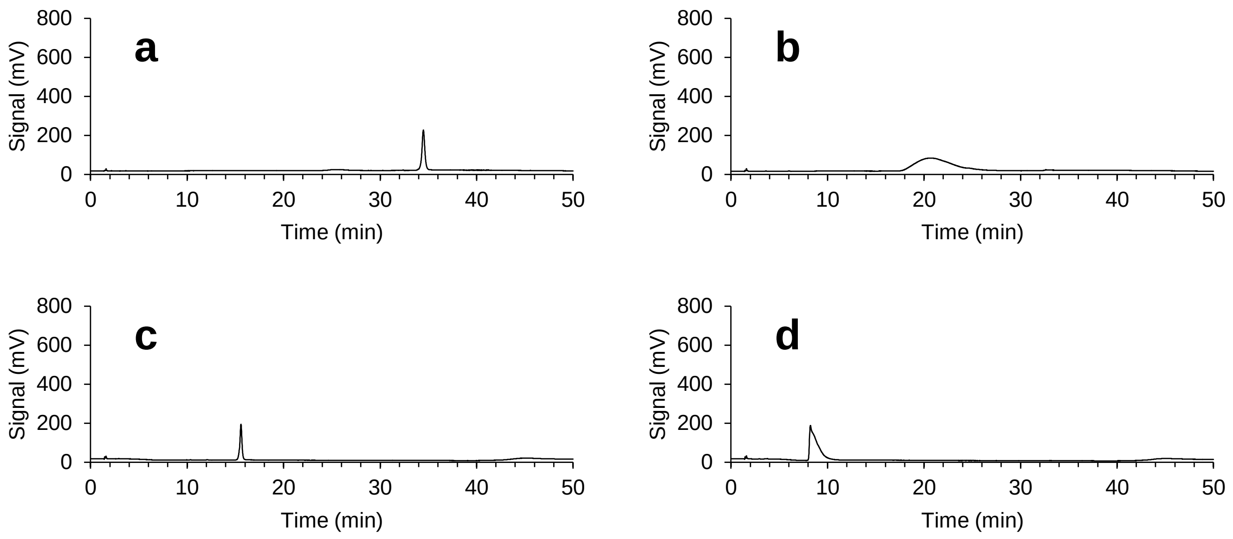


**Additional file 2: Figure S2.** Standards for HPAEC-PAD analysis. **(a)** Glucuronic acid eluted with gradient 1, **(b)** gluconic acid eluted with gradient 1, **(c)** glucuronic acid eluted with gradient 2, and **(d)** gluconic acid eluted with gradient 2.
