# Supplementary material for: Mechanisms of laccase-mediator treatments improving the enzymatic hydrolysis of pre-treated spruce
Source: Biotechnol Biofuels. 2014 Dec 24;7:177. doi: 10.1186/s13068-014-0177-8 (PMC4297466; doi:10.1186/s13068-014-0177-8)
Supplement: Additional file 3: Figure S3. — Oxygen consumption of Trametes hirsuta laccase (10 nkat ml−1) and 0.5 mM mediator: HBT (blue line), ABTS (red line), TEMPO (green line), and AS (purple line). Measured for (a) 20 min and (b) 12 h. [file 13068_2014_177_MOESM3_ESM.pdf]

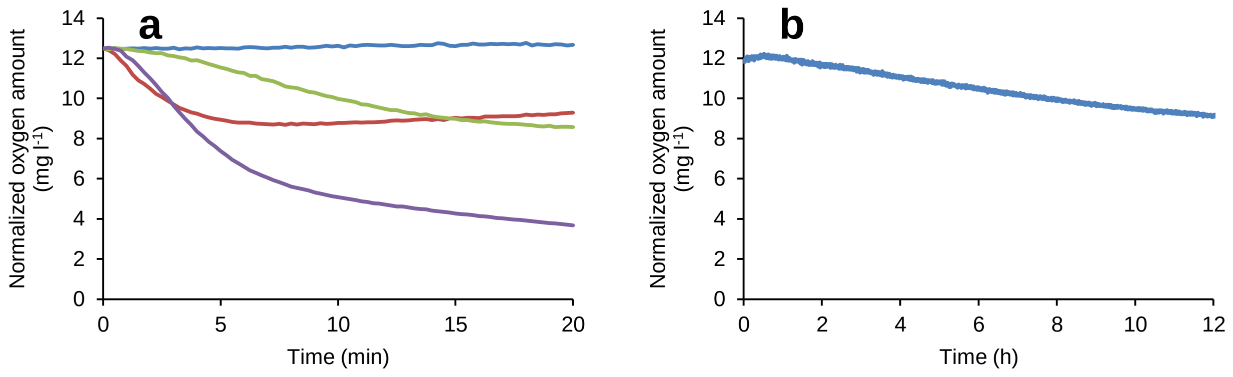

Additional file 3: Figure S3. Oxygen consumption of *Trametes hirsuta* laccase (10 nkat ml^{-1}) and 0.5 mM mediator: HBT (blue line), ABTS (red line), TEMPO (green line), and acetosyringone (purple line). Measured for **(a)** 20 min and **(b)** 12 h. Oxygen consumption was measured using FIBOX fibre-optic oxygen meter in combination with Oxy-10 10-channel minisensors (Presens Precision Sensing, Germany) using a method modified from Lahtinen et al. [1]. The reactions were carried out in 50 mM sodium citrate buffer, pH 5, under constant mixing on a magnetic stirrer at 250 rpm in sealed vials. The vials were filled with buffer, stabilized for 15 min, after which 40 µl of mediator solution was added through a septum in the cap, and stabilized for 20 min. The reaction was initiated by adding 20 µl of laccase solution.

1. Lahtinen M, Kruus K, Boer H, Kemell M, Andberg M, Viikari L, Sipilä J: **The effect of lignin model compound structure on the rate of oxidation catalyzed by two different fungal laccases**. *J Mol Catal B: Enzym* 2009, **57**:204-210.
